# Anaemia among Third-trimester Pregnant Women in a Community Hospital: A Descriptive Cross-sectional Study

**DOI:** 10.31729/jnma.7844

**Published:** 2023-01-31

**Authors:** Kamal Kandel, Bhavana Paudel, Eva Gauchan, Suraj Adhikari, Nisha Khadka, Prashant Chaulagain, Prakash Banjade, Ananta Hari Poudel

**Affiliations:** 1 Matrishisu Miteri Hospital, Pokhara, Kaski, Nepal; 2 Kathmandu Medical College, Sinamangal, Kathmandu, Nepal; 3 Department of Paediatrics, Manipal College of Medical Sciences, Pokhara, Kaski, Nepal; 4 Department of Obstetrics and Gynecology, Om Hospital and Research Centre, Chabahil, Kathmandu, Nepal; 5 Kathmandu University, Dhulikhel, Kavre, Nepal

**Keywords:** *anemia*, *maternal-child health services*, *prevalence*

## Abstract

**Introduction:**

Anaemia has haemoglobin levels of less than 11/100 ml in the first and third trimesters and less than 10/100 ml in the second. Maternal anaemia is a global health issue that has a negative impact on neonatal outcomes. The prevalence is more common in developing nations like Nepal. Positive correlations have been found between third-trimester maternal haemoglobin and neonatal birth weight. Our study aimed to find out the prevalence of anaemia among third-trimester of pregnant women in a community hospital.

**Methods:**

This was a descriptive cross-sectional study conducted in the outpatient Department of Obstetrics and Gynecology from September 2020 to September 2021. Ethical approval was taken from the Nepal health research council (Registration number: 577/2020P). The haemoglobin level of 375 participants was recorded. Data were analyzed using Statistical package for social sciences (SPSS) version 22. Convenience sampling was used. Point estimate and 95% Confidence Interval were calculated.

**Results:**

Out of a total of 375 pregnant females in the third trimester, 31 (8.27%) (5.48-11.06, 95% Confidence Interval) were anaemia.

**Conclusions:**

The prevalence of anaemia was lower as compared to other studies done in similar settings.

## INTRODUCTION

Anaemia has haemoglobin levels of less than 11/100 ml in the first and third trimesters and less than 10/100 ml in the second.^1^ Anaemia in mothers has grown to be a major global health issue. It is linked to unfavourable consequences for both the mother and the fetus, including higher maternal and perinatal mortality rates, etc.^2^

Worldwide, anaemia affects 51% of expectant mothers, and the frequency is 3-4 times higher in developing nations than in industrialized ones. In developing nations, anaemia affects 35 to 75% of pregnant women. Due to inadequate oxygenation of the placental tissue, anaemia can either be a direct cause of the decline in in-utero fetal growth or an indirect sign of inadequate maternal nutrition.

This study aimed to find out the prevalence of anaemia among third-trimester of pregnant women in a community hospital.

## METHODS

A descriptive cross-sectional study was carried out in the department of obstetrics and gynaecology at Matrishishu Miteri Hospital, Pokhara, from September 2020 to September 2021. Ethical clearance was taken from the institutional review board of the Nepal Health Research Council (Registration number: 577/2020 P). Pregnant women with singleton pregnancies in the age group of >18 to 45 years who were willing to participate in the study were included. Pregnant women who were smokers, and alcoholics, had diabetes mellitus and hypertension, babies with congenital infections and congenital malformations, intrauterine fetal demise, and dead fetuses were not included in the study. Convenience sampling was used with a self-administered questionnaire devised with the help of a literature review and consultation with the experts and advisors. The sample size was calculated using the following formula:


$$\begin{array}{ll} \text{n} & ={\text{Z}}^{2}\times \frac{\text{p}\times \text{q}}{{\text{e}}^{\text{2}}}\\ & =1.96^{2}\times \frac{0.306\times 0.694}{0.05^{2}}\\ & =324 \end{array}$$

Where,

n = minimum required sample size
Z = 1.96 at 95% Confidence Interval (CI)
p = prevalence of anaemia was 30.66% in the third trimester in the study done^3^
q = 1-p
e = margin of error, 5%

Taking the non-response rate of 10%, the sample size in the study was 375. The value of maternal haemoglobin in the third trimester was collected as per the lab report of the hospital itself (using Coulter Symex, Xp-300TM Automated Hematology Analyzer).

Data were analyzed using the IBM SPSS Statistics version 22. Point estimate and 95% CI were calculated.

## RESULTS

Among 375 pregnant females in the third trimester, the prevalence of anaemia was 31 (8.27%) (5.48-11.06, 95% Confidence Interval). Out of 31, 27 were between the ages of 20-35 years. The mean haemoglobin level was 12.04 g/dl. The maternal demographics are presented (Table 1).

**Table 1:** Maternal demographics (n= 31).

Parameters		n (%)
Age in years	<20	3 (9.68)
	20-35	27 (87.10)
	>35	1 (3.23)
Gravida	Primigravida	11 (35.48)
	Multigravida	20 (64.52)
Education	Illiterate	4 (12.90)
	Basic	21 (67.74)
	Secondary	4 (12.90)
	College	2 (6.45)
Occupation	Housewife	22 (70.97)
	Labour work	7 (22.58)
	Business	-
	Service	2 (6.45)
Knowledge	Yes	31 (100)
on ANC	No	-
Residence	Urban	25 (80.65)
	Rural	6 (19.35)

The median birth weight of neonates born to study participants was 3200 grams. Only 1% of these neonates were small for gestational age. The median gestational age was 39 weeks, and about 1% of the neonates born were preterm. The median length and head circumference of newborns was 48 cm and 34.2 cm, respectively.

## DISCUSSION

The prevalence of anaemia in the third trimester, as seen in this study, is 8%. This data suggest that anaemia during pregnancy is still a concerning issue that should be addressed. The prevalence was lower than the study done in Nepal at 30.66% and 13% prevalence.^4,5^ However, the contrast was found with the studies from other countries, Egypt at 70% and Ghana at 72.1%, suggesting a significant variance in anaemia prevalence between nations and perhaps even within a single country.^6,7^ The difference may be due to routine prenatal exams and general healthcare advancements in the community under study. Our Health centres' focus on maternal and child health may have positively impacted the lower prevalence of maternal anaemia. It is suggested that the variation may be attributed to the difference in causes, dietary habits, and population demographics.

On age-wise distribution, anaemia was higher by 27% in the age group between 25-35; however, with an increase in age, the prevalence was low. These results may be due to the enrollment of more people from the respective age group. However, the literature review also showed similar findings.^8^ Multigravida pregnant females were more anaemic than primigravida, which was comparable, with multiparty being the risk factor for anaemia.^9^

The prevalence of anaemia decreases with the mother's level of formal education. Homemakers and labour workers also make up the most considerable percentage of anaemic pregnant females. These data were congruent with national and international studies.^10^

According to our study, 15% of pregnant women who are aware of antenatal checkups were shown to be anaemic. At the same time, anaemia was observed in 25% of urban females. This can be attributed to patients' resistance to appointments and follow-up care and the fact that some ran out of iron supplementation. Action can be conducted at the hospital and community levels to raise awareness about regular antenatal checkups and iron supplementation.

This study was conducted in a single centre hospital, so these results cannot be generalized to the whole country also the study had convenience sampling, and there could have been a recording system biasing in the record of cases.

## CONCLUSIONS

The prevalence of anaemia was lower as compared to other studies done in similar settings. Maternal parity, age, education, and socioeconomic status were the major determinants of anaemia. Awareness about an antenatal checkup, enough availability of iron, and focusing more on multigravida pregnancy may likely result in decreased prevalence. Further, comparative studies between community-level healthcare centres and tertiary healthcare centres will guide the differences we observed.
